# NMDA-receptor antagonists block B-cell function but foster IL-10 production in BCR/CD40-activated B cells

**DOI:** 10.1186/s12964-014-0075-5

**Published:** 2014-12-05

**Authors:** Narasimhulu Simma, Tanima Bose, Sascha Kahlfuß, Judith Mankiewicz, Theresa Lowinus, Fred Lühder, Thomas Schüler, Burkhart Schraven, Martin Heine, Ursula Bommhardt

**Affiliations:** Institute of Molecular and Clinical Immunology, Otto-von-Guericke-University Magdeburg, Leipziger Str. 44, 39120 Magdeburg, Germany; RG Molecular Physiology, Leibniz Institute of Neurobiology, Brenneckestr. 6, 39118 Magdeburg, Germany; Department of Neuroimmunology, Institute for Multiple Sclerosis Research and The Hertie Foundation, Waldweg 33, 37073 Göttingen, Germany; Department of Immune Control, Helmholtz Centre for Infection Research, Inhoffenstr. 7, 38124 Braunschweig, Germany

**Keywords:** B cell, B10, Ifenprodil, IL-10, K_v_1.3, K_Ca_3.1, LPS, Memantine, NMDA-receptor antagonist

## Abstract

**Background:**

B cells are important effectors and regulators of adaptive and innate immune responses, inflammation and autoimmunity, for instance in anti-NMDA-receptor (NMDAR) encephalitis. Thus, pharmacological modulation of B-cell function could be an effective regimen in therapeutic strategies. Since the non-competitive NMDAR antagonist memantine is clinically applied to treat advanced Alzheimer`s disease and ketamine is supposed to improve the course of resistant depression, it is important to know how these drugs affect B-cell function.

**Results:**

Non-competitive NMDAR antagonists impaired B-cell receptor (BCR)- and lipopolysaccharide (LPS)-induced B-cell proliferation, reduced B-cell migration towards the chemokines SDF-1α and CCL21 and downregulated IgM and IgG secretion. Mechanistically, these effects were mediated through a blockade of K_v_1.3 and K_Ca_3.1 potassium channels and resulted in an attenuated Ca^2+^-flux and activation of Erk1/2, Akt and NFATc1. Interestingly, NMDAR antagonist treatment increased the frequency of IL-10 producing B cells after BCR/CD40 stimulation.

**Conclusions:**

Non-competitive NMDAR antagonists attenuate BCR and Toll-like receptor 4 (TLR4) B-cell signaling and effector function and can foster IL-10 production. Consequently, NMDAR antagonists may be useful to target B cells in autoimmune diseases or pathological systemic inflammation. The drugs’ additional side effects on B cells should be considered in treatments of neuronal disorders with NMDAR antagonists.

## Background

B cells are important mediators of the adaptive immune response by their ability to provide antigen presentation and costimulation for T cells and to differentiate into antibody secreting plasma cells. B cells are activated through the ligation of their antigen-specific B-cell receptors (BCR) and costimulatory ligands such as CD40, which drive their proliferation, survival and differentiation [1]. In addition, B cells can be stimulated by innate signals like lipopolysaccharide (LPS), a major constituent of the gram-negative bacterial cell wall that binds to Toll-like receptor 4 (TLR4) expressed on B cells [2,3]. TLR4 plays a pivotal role in the initiation of inflammation and is considered as a potent drug target to prevent severe sepsis, the leading cause of death amongst critically ill patients [4–6]. Systemic inflammation induced by LPS also seems to affect neuronal pathology, for instance in multiple sclerosis, Alzheimer’s and Parkinson’s disease [7–10].

Ligation of the BCR leads to the activation of several signaling cascades resulting in Ca^2+^-mobilization [11–13], induction of Ca^2+^/calmodulin-dependent transcription factors like NFAT [14,15] and the activation of Erk1/2 and PI-3K-Akt-mTOR signaling pathways [16–20]. The complex TLR4 signaling pathway relies on the recruitment of MyD88 and other adaptor and intermediate signaling molecules to the receptor, but ultimately also involves activation of the MAPK and Akt pathways [21–23]. Activated B cells differentiate into various B-cell subsets which contribute to a protective humoral immune response. Among them are IL-10 producing regulatory B cells (B10 cells) [24–27] which require for their formation BCR engagement and activation via the CD40 molecule or LPS stimulation [28–30]. B10 cells play a crucial role in preventing inflammatory and autoimmune pathologies [24,29,31,32] and a lack of or inhibition of B10 cells has been associated with exacerbated experimental autoimmune encephalitis (EAE) [33,34], collagen-induced arthritis [35] or colitis in mice [36]. However, B cells can also contribute to or induce diseases by production of auto-antibodies as in rheumatoid arthritis, lupus erythematosus and some neuronal disorders [7,37]. Auto-antibodies against transmitter receptors or voltage-gated ion channels in the brain influence the opening behaviour of neuronal ligand- and voltage-gated ion channels [38], leading to synaptic dysfunction, and are found in Rassmussen encephalitis [39], Lambert-Eaton myasthenic syndrome [40] or anti-N-methyl-D-aspartate-receptor (NMDAR) encephalitis [41,42]. Thus, pharmaceuticals that regulate B-cell function by modulating BCR- or TLR4-induced signaling are of interest as anti-inflammatory agents and immunotherapeutics [43,44].

NMDAR antagonists block the activity of ionotropic glutamate receptors of the NMDA type, which play a central role in synaptic transmission, memory formation and neuronal excitotoxicity [45]. NMDAR antagonists like memantine and ketamine are in use or trial to treat neuronal disorders like Alzheimer’s disease and resistant depression, respectively [46,47]. The possibility of their oral application and their non-competitive action on the channel pore, but not the glutamate binding site, make those antagonists suitable to control the glutamatergic transmission in the brain in chronic treatments of neurological diseases [48,49].

In view of the implication of B cells as source for antibodies against receptors and ion channels causing neuronal autoimmune diseases, their immune regulatory function [50] and role in LPS-induced inflammation [51], we investigated how non-competitive NMDAR antagonists modulate B-cell function. We found that the drugs impair B-cell migration, BCR- and LPS-induced proliferation and immunoglobulin (Ig) production. For both stimulatory conditions, inhibition was mediated through cross-inhibition of K_v_1.3 and K_Ca_3.1 potassium channels and attenuated B-cell signaling. However, antagonist ifenprodil could enhance the production of IL-10, fostering an anti-inflammatory B10 phenotype. Hence, non-competitive NMDAR antagonists may be suitable drugs to dampen pathological inflammatory reactions and to modulate B-cell function in autoimmune diseases. The additional effects of NMDAR antagonists on B cells may be beneficial in treating neuronal disorders.

## Results

### NMDAR antagonists block B-cell proliferation induced by BCR or LPS stimulation

**Figure 1:**
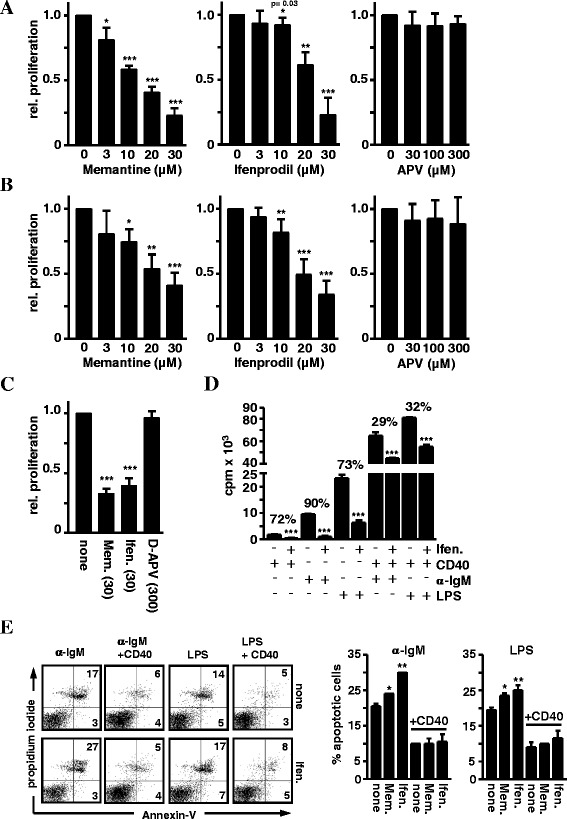
**Effects of NMDAR antagonists on B-cell proliferation and apoptosis. A-D)** NMDAR antagonists impair BCR-, TLR4- and PMA/IO-induced B-cell proliferation. Splenic B cells were stimulated with **A)** α-IgM (10 µg/ml) **B)** LPS (10 µg/ml), **C)** PMA and ionomycin (IO) or **D)** α-IgM or LPS in combination with CD40 Abs (5 µg/ml) in the presence or absence of the indicated concentrations of memantine, ifenprodil or D-APV. Proliferation was determined by ^3^[H]-Thymidine incorporation (cpm) at 24 h. Data in the graphs represent the mean + SD relative proliferation of at least two experiments. **E)** NMDAR antagonists enhance B-cell apoptosis. B cells were stimulated with α-IgM or LPS plus/minus CD40 Abs in the presence or absence of ifenprodil or memantine (30 μM each) for 24 h. Apoptosis was measured with Annexin V and propidium iodide (PI) staining and flow cytometry. The percentage of AnnexinV^+^ PI^−^ early and AnnexinV^+^PI^+^ late apoptotic cells is indicated in the dot plots (left). Data in the right graphs show the percentage of apoptotic cells as mean + SD calculated from two experiments.

Splenic B cells were stimulated with anti-IgM (Fab’)_2_ fragment goat anti-mouse (α-IgM) to mimic BCR triggering by antigens, or with the TLR4 ligand LPS. B-cell proliferation was determined by ^3^[H]-Thymidine incorporation at 24 h in the presence or absence of the NMDAR antagonists memantine, an NMDAR open-channel blocker, ifenprodil, a non-competitive inhibitor of the GluN2B subunit of NMDARs, or the competitive NMDAR antagonist D-APV [49,52]. Memantine and ifenprodil inhibited α-IgM- as well as LPS-induced DNA synthesis in a concentration dependent manner (Figure 1A and B). In contrast, the competitive antagonist D-APV had no effect, even at very high doses (300 μM). The proliferative response of B cells activated with PMA and ionomycin (IO) was also inhibited by ifenprodil and memantine, but not by D-APV (Figure 1C). Costimulation by CD40 Abs enhanced α-IgM- and LPS-induced B-cell proliferation, and under these conditions the antagonists only had a weak inhibitory effect, reducing DNA synthesis by 29-32%, respectively, compared to a 72-90% reduction in the absence of CD40 stimulation (Figure 1D).

The effects of NMDAR antagonists on apoptosis was evaluated on B cells activated for 24 h with α-IgM, α-IgM + CD40, LPS, and LPS + CD40 (Figure 1E). 5-10% more apoptotic cells were detected in antagonist-treated cultures whereby ifenprodil had stronger effects than memantine, especially on B cells stimulated with α-IgM only. In case of CD40 costimulation, B-cell apoptosis was much lower and both antagonists had no enhancing effect on cell death.

### NMDAR antagonists induce membrane depolarization and inhibit K_v_1.3 and K_Ca_3.1 channels in B cells

**Figure 2:**
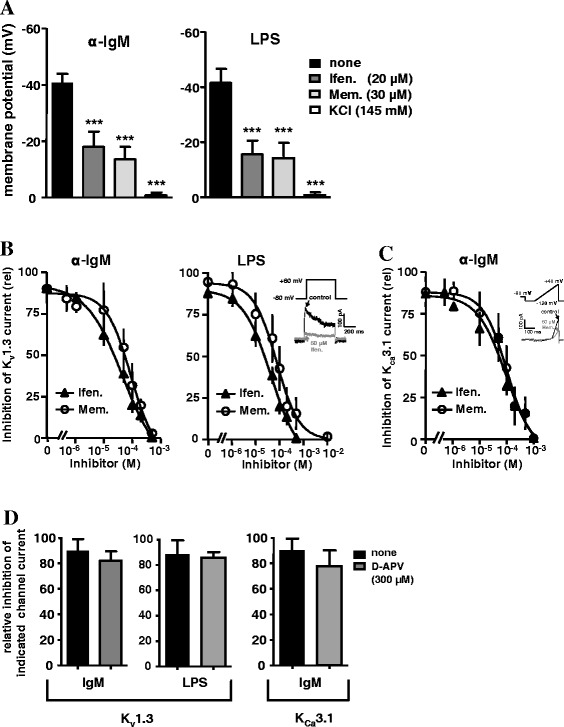
**Effects of NMDAR antagonists on B-cell membrane potential and K** ^**+**^ **channel activity.** B cells were activated with α-IgM or LPS (10 μg/ml each) for 24–48 h. **A)** NMDAR antagonists lead to a depolarization of the membrane potential. Activated B cells were analyzed for changes in the membrane potential upon addition of ifenprodil or memantine in concentrations as indicated. KCl treatment served as a control for cell integrity. **B-D)** Ifenprodil and memantine inhibit K^+^ channel activity. Dose**-**inhibition curves of **B)** K_v_1.3 and **C)** K_Ca_3.1 channels in the presence of ifenprodil or memantine were plotted from the recorded maximal transient currents. Insets show one particular trace of control and inhibiting current along with the protocol used for measuring **B)** K_v_1.3 and **C)** K_Ca_3.1 channels. **D)** Data in the graphs represent the relative inhibition of K_v_1.3- and K_Ca_3.1-mediated currents in the presence of the competitive NMDAR antagonist D-APV. All data were calculated from 5–6 cells of four experiments and are represented as mean ± SD.

We previously reported that protein expression of functional NMDARs in murine T cells is elusive and that NMDAR antagonists inhibit K_v_1.3 and K_Ca_3.1 channels [53], which are considered as potent targets for immunosuppression [54,55]. These potassium channels are also expressed on B cells and their inhibition was found to differentially influence B-cell proliferation after BCR activation or PMA/IO stimulation [56–59]. Since K_Ca_3.1 and K_v_1.3 channel activities influence membrane depolarization and, thereby, the Ca^2+^-flux into the cell [60], we first determined the drugs’ effects on the membrane potential. Ifenprodil (20 μM) and memantine (30 μM) reduced the membrane potential of α-IgM- or LPS-activated B cells from ~ −40 mV to ~ −20 mV and ~ −10 mV, respectively. Addition of KCl served as a positive control for membrane depolarization (Figure 2A). Next, we recorded K_v_1.3 channel-mediated currents from activated B cells and the dose response curves in the presence of inhibitors were calculated from maximal transient current amplitudes. Ifenprodil and memantine markedly reduced K_v_1.3 channel currents irrespective whether B cells were stimulated with α-IgM or LPS (Figure 2B). IC_50_ and Hill slope values for α-IgM-activated B cells were ~20 μM and ~1.3 for ifenprodil and ~40 μM and ~1.8 for memantine. For LPS-treated B cells, IC_50_ and Hill slope values were ~18 μM and ~1.4 for ifenprodil and ~45 μM and ~1.2 for memantine. For B cells stimulated by BCR ligation, we additionally recorded K_Ca_3.1 channel-mediated currents (Figure 2C). K_Ca_3.1 currents were not detected in LPS-activated B cells. IC_50_ values for ifenprodil and memantine were ~30 μM and ~50 μM and Hill slopes were ~1.4 and ~1.6. However, the competitive NMDAR antagonist D-APV, which blocks neuronal NMDARs at the 1 μM range, had no effect on K_v_1.3 and K_Ca_3.1 channels, even at 10-time higher concentrations (300 μM) (Figure 2D). Thus, K_v_1.3 and K_Ca_3.1 channels, whose specific blockade abolishes B-cell activation [56,59], are partially inhibited by the non-competitive NMDAR antagonists ifenprodil and memantine.

### BCR- and LPS-induced B-cell signaling is attenuated by NMDAR antagonist

**Figure 3:**
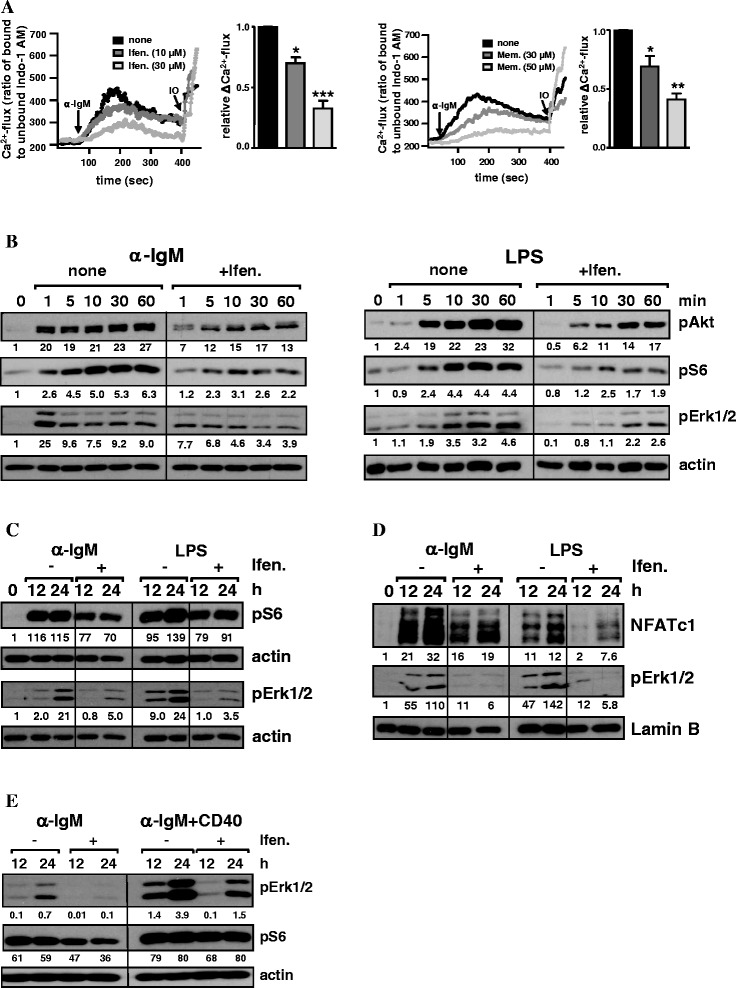
**Effects of NMDAR antagonists on B-cell signaling. A)** Reduced Ca^2+^-flux in BCR-activated B cells in the presence of NMDAR antagonists. Indo-1 AM-labelled B cells were stimulated with α-IgM (10 μg/ml) in the presence or absence of ifenprodil (left) or memantine (right) and Ca^2+^-flux was determined by flow cytometry. Corresponding graphs show the mean + SD relative ΔCa^2+^-flux of three experiments. **B-E)** NMDAR antagonists attenuate BCR- and LPS-induced activation of important signaling molecules. B cells were activated with α-IgM (10 µg/ml) or LPS (10 µg/ml) or α-IgM plus CD40 Abs (5 µg/ml) in the presence or absence of ifenprodil (30 μM) in **B)** short-term and **C-E)** long-term stimulation. Activation of the indicated signaling proteins in **B)** total, **C, E)** cytoplasmic and **D)** nuclear protein extracts was analyzed by Western blot. β-Actin and Lamin B expression served as controls for protein loading. Indicated numbers give the relative protein expression after quantification and normalization to controls. Data are the representative of two **(E)** and three **(B-D)** independent experiments.

Next, we assessed the antagonists’ effects on B-cell signaling and Ca^2+^mobilization, which is critical for B-cell activation and proliferation [11,13,61]. Indo-1 AM-labelled B cells showed a concentration-dependent inhibition of BCR-induced Ca^2+^-flux upon treatment with ifenprodil or memantine (Figure 3A). Furthermore, the levels of phosphorylated Akt, S6 and Erk1/2 were significantly lower in α-IgM-activated B cells in the presence of ifenprodil compared to untreated cells (Figure 3B, left panel). Notably, B cells stimulated with LPS showed a very similar inhibition of Akt, S6 and Erk1/2 activation by ifenprodil (Figure 3B, right panel). In long-term stimulation, α-IgM- and LPS-activated B cells cultured with ifenprodil exhibited lower levels of pErk1/2 and pS6 in the cytoplasm (Figure 3C) and a reduced nuclear accumulation of pErk1/2 and NFATc1 (Figure 3D). Thus, NMDAR antagonists downregulate major signaling events of two distinct B-cell activating receptors that play an important role in innate and antigen-specific B-cell responses [18,62]. Since CD40 costimulation rescued the inhibitory effects of NMDAR antagonists on BCR-induced B-cell proliferation and apoptosis (Figure 1), we analysed pErk1/2 and pS6 expression under costimulatory conditions and found an enhanced activation of both signaling molecules compared to α-IgM stimulation alone (Figure 3E). However, although addition of ifenprodil reduced pErk1/2 and pS6 levels in α-IgM + CD40-treated B cells, these levels were still above those found after α-IgM treatment. Hence, antagonist-induced attenuated signaling in CD40 costimulated B cells is still above a critical threshold needed for B-cell activation.

### NMDAR antagonists impair B-cell migration and Ig production

**Figure 4:**
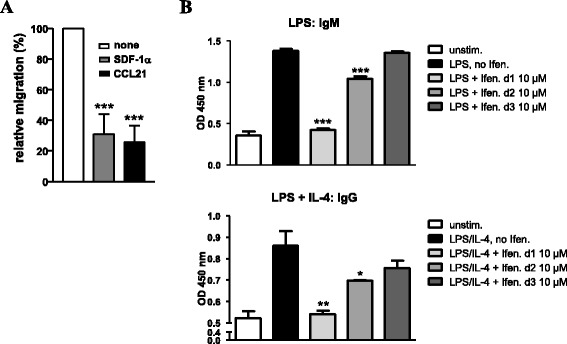
**NMDAR antagonists affect B-cell migration and Ig production. A)** Ifenprodil impairs chemokine-induced B-cell migration. The migration of splenic B cells towards SDF-1α and CCL21 in the absence or presence of ifenprodil (30 μM) was determined and the relative + SD migration was calculated from three experiments. Migration of B cells in the absence of ifenprodil was set as 100%. **B)** Ifenprodil blocks Ig production. B cells were activated with LPS or LPS plus IL-4 in the presence or absence of ifenprodil (10 μM) and IgM and IgG secretion was determined at day 4 with ELISA. Ifenprodil was added at day 1, day 2 or day 3. Data are representative for two experiments.

The migratory response of B cells within the activating lymphoid environment or at inflammatory sites is a key feature for their differentiation and function. We investigated whether NMDAR antagonists affect chemokine-induced migration and found a strong reduction in the migratory response of B cells to the chemokines SDF-1α and CCL21 in the presence of ifenprodil (Figure 4A). Antibody secretion is the major effector function of B cells. In order to determine the impact of ifenprodil on IgM and IgG production, B cells were stimulated with LPS or LPS + IL-4. Ifenprodil was added at days 1, 2 or 3 and ELISA was performed at day 4. As shown in Figure 4B, the blockade of IgM and IgG secretion was most efficient after addition of ifenprodil at day 1. With increasing time, the inhibitory effect of ifenprodil declined but was still detectable. Hence, NMDAR antagonists not only inhibit B cell proliferation and migration but also antibody secretion.

### NMDAR antagonists modulate IL-10 production

**Figure 5:**
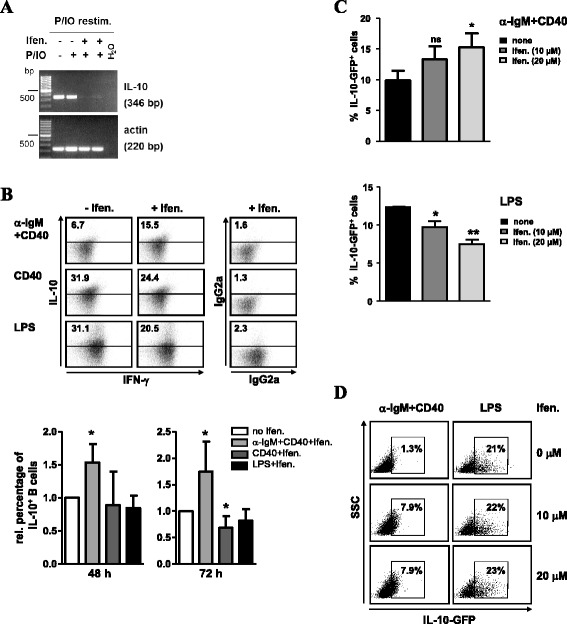
**Ifenprodil modulates IL-10 production of B cells. A)** Ifenprodil reduces IL-10 mRNA transcripts in PMA/IO-activated B cells. Splenic B cells were left untreated or were activated with PMA and IO in the absence or presence of ifenprodil (10 and 30 μM, lanes 3 and 4) for 16 h. Cells were restimulated with PMA/IO and monensin for 6 h and RT-PCR for IL-10 and β-actin expression was performed. Data are the representative of two experiments. **B-D)** IL-10 production in BCR + CD40-stimulated B cells is enhanced upon ifenprodil treatment. **B)** B cells were activated with α-IgM + CD40, CD40 Abs alone or LPS for 2–3 days. Ifenprodil (10 μM) was added at day 1. The production of IFN-γ and IL-10 was analyzed by intracellular staining and flow cytometry. The percentage of cells positive for IL-10 (gates were set according to staining with isotype controls, right panel) is indicated in each dot plot. The graphs represent the relative + SD percentage of IL-10 expressing B cells in α-IgM + CD40, CD40 and LPS cultures treated with ifenprodil. For each stimulation condition, data were related to the percentage of IL-10^+^ B cells generated in the absence of ifenprodil, which was set as 1.0. Data for 48 h were calculated from three, for 72 h from two to four experiments. **C**, **D)** B cells from IL-10-GFP tiger mice were activated with α-IgM+CD40 or LPS. Ifenprodil (10 and 20 μM) was added after 21-25 h and expression of IL-10-GFP at **C)** day 2 or **D)** day 4 was determined with flow cytometry. Data are from two independent experiments.

Since several B-cell responses were negatively regulated by NMDAR antagonists, we asked whether the drug-induced attenuated signaling would influence the production of IL-10, the immunosuppressive cytokine made by B10 cells [18,32,33,35]. Mitogenic stimulation of B cells with PMA and IO leads to the induction of IL-10 mRNA [24,31,63]. Thus, we stimulated B cells with these mitogens for 16 h in the absence or presence of ifenprodil. Drug treatment lead to a strong repression of IL-10 transcripts compared to untreated B cells (Figure 5A). We then asked whether ifenprodil has effects on B cells that were pre-activated with α-IgM + CD40 Abs, LPS or agonistic CD40 Abs, which are known to give rise to regulatory B10 cells [25,28,35,64]. Ifenprodil was added at day 1, and IL-10 and IFN-γ production were determined at day 2 or 3 (Figure 5B). IFN-γ production was not altered by ifenprodil. Low levels of IL-10 production were induced in 8% of α-IgM + CD40-stimulated and in 19-27% of CD40- or LPS-activated B cells, which showed low to high levels of IL-10. Ifenprodil had either no effect or lowered the percentage of IL-10 producing B cells in CD40- or LPS-stimulated cultures. In contrast, addition of ifenprodil to α-IgM + CD40-activated B cells increased the frequency of IL-10 producers 1.5-2-fold, although absolute IL-10 expression levels remained low. Experiments with B cells from IL-10-GFP knock-in tiger mice [65] supported these results. α-IgM + CD40-activated B cells, with ifenprodil treatment started after 21–25 h, showed a 50% increase in the percentage of IL-10-GFP^+^ B cells at day 2 (Figure 5C) and when measured at day 4 a 6-fold increase (Figure 5D). Therefore, ifenprodil can foster the generation of an IL-10 producing phenotype.

## Discussion

Here, we show that non-competitive NMDAR antagonists attenuate adaptive (BCR) as well as innate (LPS/TLR4) B-cell signaling. The drugs inhibited IgM and IgG secretion, B cell migration and impaired B-cell proliferation and viability, which were partially overcome by CD40 costimulation. Since the non-competitive antagonists ifenprodil and memantine, but not the competitive antagonist D-APV, blocked the activity of K_v_1.3 and K_Ca_3.1 channels, the used non-competitive antagonists seem to act mainly via inhibition of those K^+^ channels, which maintain a favourable electrochemical gradient that is required for a sustained Ca^2+^-entry through Ca^2+^-release activated channels (CRAC) [15,66]. Due to the similar action of the non-competitive NMDAR antagonists on K_v_1.3 and K_Ca_3.1 channels as described for the action of specific K^+^ channel inhibitors [57–59], a differential modulation seems excluded and is probably the cause for their effects reported here. In line with the inhibition of both K^+^ channel types in B cells, BCR-induced Ca^2+^-flux was reduced and BCR- and TLR4-induced downstream activation of Erk1/2, Akt, S6, and NFATc1 was dampened. Ifenprodil added to B cells pre-activated with BCR/CD40 Abs fostered IL-10 production, but when added at the beginning of B-cell stimulation reduced IL-10 transcripts. Thus, the enhancement of IL-10 production seems to depend on the timing and concentration of drug application, although further experimentation is required for identification of the underlying mechanisms. IL-10 expression is regulated by Ca^2+^-level [63], Bruton’s tyrosine kinase (Btk), the adaptor protein BLNK/SLP65, CamKII, Erk1/2, and transcription factors like CREB, STAT3, NFκB or NFAT [13,18,67–72]. The activation of Erk1/2 in B cells in turn is dependent on Ca^2+^-flux and PI-3K activation [73,74]. IL-10 secretion is differentially controlled depending on the activation stimulus and availability of IL-21 [26,75]. Furthermore, IL-10 production by B10 cells seems to be transient [76]. We found that ifenprodil impairs BCR/CD40-induced Erk1/2 and Akt activation and thus speculate that upon ifenprodil treatment subtle differences in Ca^2+^-level [77] and the activity of Erk1/2, Akt and other signaling molecules favour IL-10 production. Interestingly, genetic deletion or inhibition of K_v_1.3 channels in T cells was found to increase IL-10 production in T cells along with an amelioration of experimental autoimmune encephalomyelitis and allergic asthma [78,79]. Since NMDAR antagonists block K_v_1.3 and K_Ca_3.1 channels in B cells, the increase of IL-10 production in BCR/CD40-activated B cells may result from similar mechanisms.

Our data suggest that application of non-competitive NMDAR antagonists during chronic treatments of neurological disorders like Alzheimer`s disease may not only involve neuronal NMDARs, but may also have additive side-effects by targeting B cells, which are assumed to contribute to these disorders [7,9,10]. Given that the drugs impaired several B-cell functions, but enhanced IL-10 production in BCR/CD40-stimulated B cells, their employment in systemic inflammation or autoimmune diseases, for instance in sepsis or anti-NMDAR encephalitis, appears promising. Here, antagonists may limit B-cell hyper-reactivity and antibody production or mediate immunomodulation or suppression through an enhanced frequency of IL-10 secreting B cells. IL-10 producing B cells also target T cells as they induce IL-10 producing CD4^+^ T cells, suppress Th1 cell differentiation and increase the number of CD4^+^CD25^+^Foxp3^+^ regulatory T cells *in vivo* [29]. Furthermore, although action of non-competitive NMDAR antagonists on memory B cells is not investigated, pharmacological modulation of memory B-cell differentiation or secondary B-cell responses can be envisaged. Since specific blockade of K_v_1.3 and K_Ca_3.1 channels results in immunosuppression of T and B cells [54,59] and non-competitive NMDAR antagonists block these two K^+^ channels in B cells, application of NMDAR antagonists may also be useful to treat acute and chronical allograft rejections driven by B cells. Memantine, which passed clinical trials and is in use to treat advanced Alzheimer`s disease, might show similar effects as the specific K_v_1.3 and K_Ca_3.1 blockers Shk and TRAM-34 in treating allograft vasculopathy or kidney allograft rejection [80,81]. However, further studies are required to determine the drug’s suitability for *in vivo* treatment of these immune disorders.

## Conclusions

Through their nonspecific action on K_v_1.3 and K_Ca_3.1 potassium channels, non-competitive NMDAR antagonists are potent modulators of LPS/TLR4- and BCR-induced proliferation, migration, Ig production and anti-inflammatory IL-10 production by B cells. Thus, they may be useful to target B cells under pathological inflammatory conditions. They may also have beneficial side effects during chronic treatments of neurological disorders like Alzheimer’s disease.

## Methods

### Mice

C57BL/6 mice were used at the age of 6–10 weeks. IL-10-GFP knock-in mice, designated interleukin-ten ires gfp-enhanced reporter (tiger) mice [65] were 8 or 28 weeks old and kindly provided by J. Hühn, HZI Braunschweig, Germany. All animal work performed was in compliance with the German and local guidelines for the Use of Experimental Animals.

### Cell isolation and proliferation assay

Splenic B cells were isolated with the B-cell isolation kit from Miltenyi Biotech (Bergisch Gladbach, Germany) according to the manufacturer’s protocol. Purity of B cells was 90-95%. B cells were activated with α-IgM (10 μg/ml, Jackson Immunoresearch Laboratories, Hamburg, Germany), lipopolysaccharide (LPS, 10 μg/ml, E. coli 0111:B4, Sigma-Aldrich, Taufkirchen, Germany), or PMA (100 ng/ml, Calbiochem, Darmstadt, Germany) and IO (700 ng/ml, Sigma) in complete RPMI1640 medium (Biochrom AG, Berlin, Germany) supplemented with 10% FCS, 50 μM β-mercaptoethanol, 1% penicillin/streptomycin. NMDAR antagonist ifenprodil, memantine, or D-APV (diluted in ddH_2_O, all from Tocris Biosciences, Bristol, Great Britain) were added in concentrations as given. Proliferation was measured at 24 h of culture by ^3^[H]-Thymidine incorporation (0.2 μ Ci/well, MP Biochemicals Europe, Heidelberg, Germany) for 16 h.

### Apoptosis measurement

Apoptosis was determined with the Apoptosis detection kit from BD Pharmingen (Heidelberg, Germany). 2×10^5^ splenic B cells were left untreated or were activated with α-IgM (10 μg/ml) or LPS (10 μg/ml) without or with costimulation by CD40 Abs (5 μg/ml, Biolegend, San Diego, CA, USA) in the presence or absence of ifenprodil (30 μM, Tocris Biosciences). At 24 h of culture cells were harvested, stained with Annexin V-FITC (BD Pharmingen) and propidium iodide (PI, Sigma-Aldrich) according to manufacturer’s protocol and analyzed by flow cytometry using a FACSFortessa and Cell Quest software (BD Biosciences). The percentage of viable cells was determined by gating on AnnexinV^−^PI^−^ cells.

### Western blot

5×10^6^ splenic B cells were activated with α-IgM (10 μg/ml), LPS (10 μg/ml) or α-IgM (10 μg/ml) plus CD40 Abs (5 μg/ml) in the presence or absence of ifenprodil (30 μM) for the indicated time points. Cells were lysed and total, cytoplasmic or nuclear protein extracts were obtained as described before [82]. Protein lysate (10–15 μg) was subjected to 8-10% SDS-PAGE and proteins were transferred onto nitrocellulose membrane, which was blocked with 5% non-fat milk powder in TBST. Primary Abs for the detection of signaling proteins were: pErk1/2 (Thr^202^/Tyr^204^), pAkt (Ser^473^, DE9), pS6 (S^240/244^) (all from Cell Signaling Technology, Frankfurt, Germany), NFATc1 (7A6, Alexis Biochemicals, Lörrach, Germany), β-actin (AC 40, Sigma-Aldrich), and Lamin-B (Santa Cruz, Biotechnology, Santa Cruz, CA, USA). HRP-coupled mouse anti-rabbit, goat anti-mouse or donkey anti-goat secondary Abs (Jackson ImmunoResearch Laboratories, Dianova) and the ECL detection system (Thermo Scientific Pierce, Rockford, IL, USA) were applied to reveal primary antibodies. Quantification of immune reactive bands was done with Kodak software.

### Ca^2+^-flux measurement

Splenocytes were stained with Indo-1 AM (4 μM, Life Technologies, Darmstadt, Germany) for 45 min at 37°C. Cells were washed, stained for CD8 and CD4 surface expression and suspended in Hank’s buffer (Biochrom) supplemented with 1 mM CaCl_2_. NMDAR antagonists ifenprodil (10 or 30 μM) and memantine (30 or 50 μM) were added for 5 min before B cells were activated with α-IgM (10 μg/ml) to induce Ca^2+^-flux. Ionomycin (IO, 2 μM, Calbiochem) was added at the end to control cell reactivity. Ca^2+^-flux was measured with a LSRII flow cytometer (BD Biosciences). Data were analyzed with FlowJo V3.6.1 software (Tree Star, Ashland, OR, USA), mean Ca^2+^-flux of unlabelled B cells was calculated and data were further processed by IgorPro5.04B software (WaveMetrics Inc., Portland, OR, USA). ΔCa^2+^-flux represents the difference between the maximum and minimum values of Ca^2+^-intensity.

### Migration assay

Splenocytes (4×10^6^) were left untreated or were incubated with ifenprodil (30 μM) for 30 min in D-MEM medium (Biochrom) supplemented with 0.1% BSA and 10 mM HEPES pH7.4. Cells were transferred unto fibronectin-coated (6.5 μg/ml, Roche Diagnostics, Basel, Switzerland) transwell chambers (3.0 μm pore size, Corning Costar, Tewksburry, MA, USA) and SDF-1α (100 ng/ml) or CCL21 (300 ng/ml, both from PeproTech, Hamburg, Germany) was added. Migration was performed for 150 min at 37°C and stopped with 0.1 M EDTA. Migrated cells were stained with rat anti-mouse B220-FITC Ab (RA3-6B2, BD Pharmingen) and measured for 30 s at a FACS Fortessa. The number of B cells migrated in the presence of chemokine (set as 1.0) was divided by the number of cells migrated in the absence of chemokine to obtain the relative migration values.

### Intracellular cytokine staining and IL-10-GFP induction

Splenic B cells were stimulated with α-IgM (10 μg/ml) plus CD40 Abs (5 μg/ml), CD40 Abs alone (5 μg/ml), or LPS (10 μg/ml) for 48 h or 72 h. Ifenprodil (10 μM) was added once and at day 1. Before harvest, cells were treated with IO (800 ng/ml) and PMA (500 ng/ml) for 4 h in the presence of Brefeldin A (3 μg/ml, all from Calbiochem). Thereafter, cells were fixed and stained with IL-10-PE and IFN-γ-FITC Abs using IgG2b-PE/FITC isotype controls (all from eBiosciences, Frankfurt, Germany) and the FoxP3 staining buffer kit (eBiosciences) according to manufacturer’s protocol. Cells were analyzed by flow cytometry and the percentage of live cells producing IL-10 or IFN-γ was determined with Cell-Quest Pro. B cells isolated from IL-10-GFP tiger mice were activated with α-IgM/CD40 or LPS and cultured in 96-well plates for 2 or 4 days. Ifenprodil (10 and 20 μM) was added at 21–25 h and cells were harvested either at day 2 or day 4. Before harvest, cells were re-stimulated with PMA (100 ng/ml) and IO (800 ng/ml) and monensin (10 μg/ml) for 4 h. IL-10-GFP expression was analyzed on gated live cells with flow cytometry.

### Electrophysiology

For all experiments the whole-cell configuration of the patch-clamp technique was applied at room temperature (RT) (20-24°C) using an EPC10 amplifier and PatchMaster v.2.11 (HEKA Electronic, Lambrecht, Germany). Patch pipettes from borosilicate glass used for recordings had a resistance between 3–5 MΩ. For recording K_v_1.3 currents the external solution contained 145 mM NaCl, 5 mM KCl, 10 mM HEPES, 1 mM MgCl_2_, 2.5 mM CaCl_2_, 5.5 mM glucose, pH7.4 (NaOH). The pipette solution contained 140 mM KF, 11 mM EGTA, 10 mM HEPES, 1 mM CaCl_2_, 2 mM MgCl_2,_ pH7.2 (KOH) [83]. In both cases osmolarity was set to 300–340 mOsM. K_v_1.3 currents were measured with depolarizing voltage steps up to +60 mV from a holding potential of −80 mV every 30 s and sampling rate of 5 kHz. K_Ca_3.1 channel currents were measured with an external solution composed of 160 mM Na-aspartate, 4.5 mM KCl, 2 mM CaCl_2_, 1 mM MgCl_2_, 10 mM HEPES and an internal solution of 145 mM K-aspartate, 8.5 mM CaCl_2_, 2 mM MgCl_2_, 10 mM EGTA, 10 mM HEPES adjusted to pH7.4 and pH7.2, respectively. This current was recorded by a 200 ms voltage ramp from −120 to +40 mV from a holding potential of −80 mV every 15 s. For membrane potential experiments, cells were recorded in the current clamp mode with 0 pA holding current immediately after establishment of the whole-cell configuration. Ifenprodil, memantine or D-APV (Tocris) were added during the recording with a constant inhibitor concentration. Transient currents were analyzed in HEKA FitMaster v.2×53 and GraphPad Prism 5.0 to determine the dose–response curve and Hill slope and statistical analysis.

### RNA isolation and RT-PCR

Splenic B cells were stimulated with PMA (100 ng/ml) and IO (700 ng/ml) for 16 h in the presence or absence of ifenprodil (20 and 30 μM) or were left unstimulated. Before harvest cells were re-stimulated for an additional 6 h with PMA and IO and monensin (10 μg/ml). RNA was extracted with TRIzol reagent (Life Technologies, Darmstadt, Germany) and reverse transcribed with a First-Strand cDNA Synthesis Kit (Thermo Scientific, Karlsruhe, Germany) according to the manufacturer’s protocol. PCR primers were: IL-10: forward 5’-TGCCTTCAGTCAAGTGAAGACT-3’ and reverse 5’-AAACTCATTCATGGCCTTGTA-3’ and β-actin: forward 5´-CCAGGTCATCACTATTGGCAAGGA-3 and reverse 5`-GAGCA GTAATCT CCTTCTGCATCC-3’.

### ELISA

For detection of secreted IgM and IgG, B cells were activated with LPS (10 μg/ml) or LPS plus IL-4 (20 ng/ml, ImmunoTools, Friesoythe, Germany) in triplicates in 96-well plates (Nunc Maxisorp, Thermo Fisher-Scientific, Marietta, OH, USA). Ifenpodil (10 μM) was added at day 1, day 2 or day 3 and culture supernatant was taken on day 4. Plates were coated over night with 50 μl goat anti-mouse Ig (Southern Biotech, Birmingham, AL, USA) 1:500 in 50 mM sodium carbonate puffer. After washing with PBS/0.05% Tween 20 the wells were blocked with PBS/5% BSA for 1 h. The samples were diluted in PBS/5% BSA and incubated for 2 h at RT. After washing, POD-coupled anti-mouse IgM or IgG (Sigma-Aldrich, Taufkirchen, Germany) were added at 1:250 in PBS/5% BSA for 1 h at RT, followed by substrate development with TMB reagent (BD Biosciences). OD at 450 nm was determined with an ELISA reader (Sunrise, Tecan, Männedorf, Switzerland).

### Statistical analysis

Data are given as mean values ± standard deviation (SD). Student’s *t* test was used to determine statistical significances, with *p < 0.05, **p < 0.01 and ***p < 0.001.

## Abbreviations

ifen.: Ifenprodil
IO: Ionomycin
mem.: Memantine
NMDAR(s): N-methyl-D-aspartate receptor(s)
